# Postradiation extragenital lichen sclerosus of the breast

**DOI:** 10.1016/j.jdcr.2025.08.005

**Published:** 2025-08-20

**Authors:** Nadia Talebi, McKenzie E. Maloney, Rachel C. Falkner

**Affiliations:** a Medical College of Georgia, Augusta University, Augusta, Georgia; b Pinnacle Dermatology, Bluffton, South Carolina

**Keywords:** EGLS, extragenital lichen sclerosus, lichen sclerosus, LS, oncodermatology, radiation, radiation complication

## Introduction

Lichen sclerosus (LS) is a chronic mucocutaneous inflammatory disease commonly affecting the genital and perianal skin, presenting as atrophic, white papules and plaques. Patients often experience pruritus and discomfort in affected areas and can progress to adhesions, causing significant morbidity. LS is more common in females than males and affects approximately 1 in 300 to 1000 people.^1^ Diagnosis of LS is often clinical but can be confirmed with dermoscopic or histologic findings. Untreated LS may lead to scarring, dyspareunia, and potentially the development of squamous cell carcinoma.

Extragenital lichen sclerosus (EGLS), a subset of LS patients, comprises 15% to 20% of cases. The morphology of EGLS is identical to anogenital LS and presents with pruritus. However, the distribution involves the neck, proximal limbs, and upper torso. EGLS can be classified based on the extent, location, lesional morphology, and pattern.^2^ The pathophysiology of LS and EGLS is not fully understood. EGLS has been associated with various autoimmune disorders, hormonal and genetic factors, drugs, infections, and trauma.^3^ However, few cases have identified radiation therapy as a trigger.

## Case

**Fig 1:**
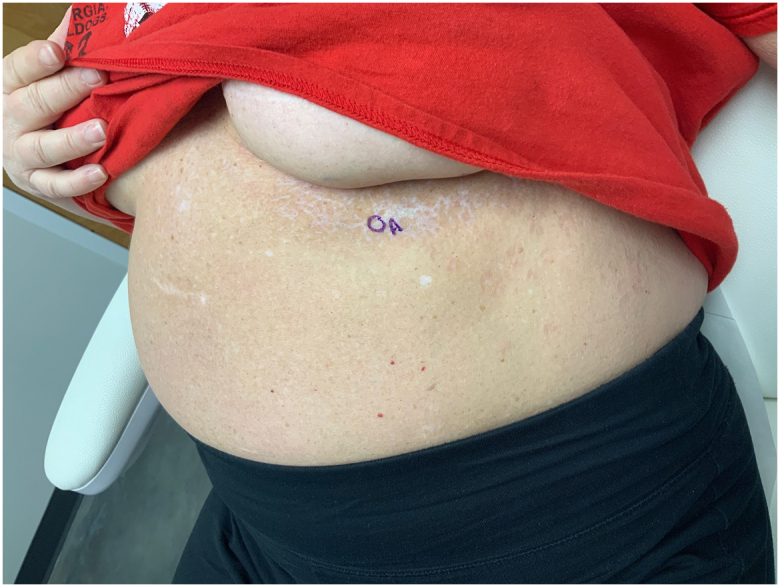
White papules coalescing into plaques on the inframammary fold.

A 58-year-old Caucasian female, with a past medical history of breast cancer status post-lumpectomy with radiation 11 years ago, presented to dermatology for unilateral, reddish-pink scaling plaques and papules involving the previously radiated inframammary area of the left chest. The lesion had been present for 2 years. She had not experienced any prior radiation-induced skin changes. On exam, there was a 5 by 7 cm hypopigmented, reddish-pink scaling plaque with overlying excoriation and lichenification (Fig 1). This plaque appeared as scattered coalescing atrophic plaques, resembling a pattern similar to “confetti-like” macules. The differential diagnosis included EGLS, morphea, systemic sclerosis, and vitiligo. Additionally, she had a history of genital LS that was clinically diagnosed by her obstetrician years before radiation and managed intermittently with topical clobetasol. At the time of presentation, she was in clinical remission, with faint atrophic vulvar plaques and minimal inflammation on exam, consistent with inactive disease.

**Fig 2:**
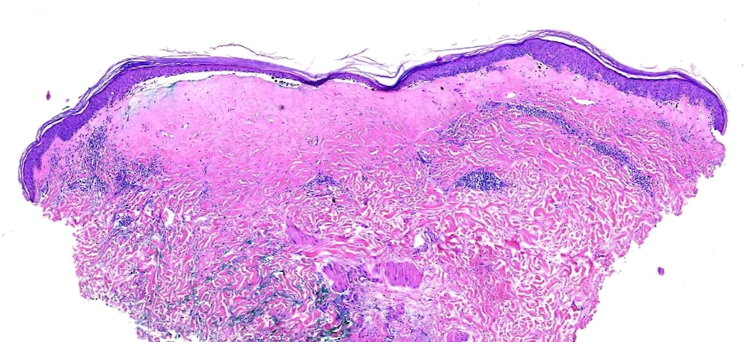
Punch biopsy (H&E stain, magnification) from the left inframammary breast showing atrophic epidermis with underlying dense homogenized collagen and inflammation. *H&E*, Hematoxylin & eosin.

**Fig 3:**
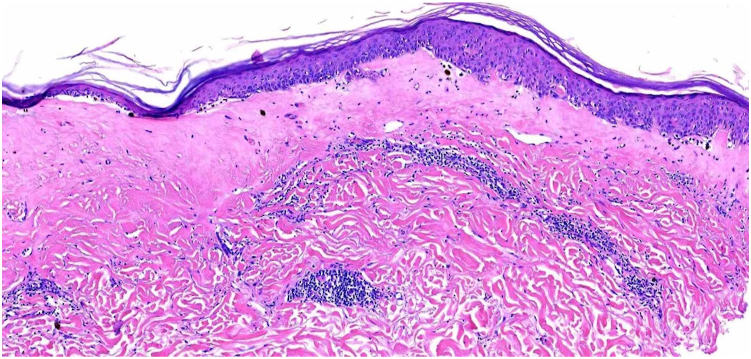
Punch biopsy (H&E stain; magnification) showing degenerative vacuolar changes in the basal keratinocytes associated with secondary subepidermal separation and pigment incontinence. *H&E*, Hematoxylin & eosin.

A 4 mm left inframammary chest punch biopsy was obtained, and the patient was prescribed a 30 g tube of topical clobetasol 0.05% ointment to apply twice daily for 4-6 weeks or until the lesion cleared. Histopathology revealed an atrophic epidermis with underlying dense homogenized collagen and vacuolar changes in basal keratinocytes associated with subepidermal separation and pigment incontinence. In the dermis, inflammatory cells and ectatic capillaries were present and downwardly displaced by the collagen, all of which are consistent with a diagnosis of LS (Figs 2 and 3). The patient did not follow up after initial treatment.

## Discussion

EGLS is a rare subtype of LS. While the pathophysiology of LS is poorly understood, it is associated with autoimmune disorders, hormonal and genetic factors, drugs, infections, and trauma; however, few cases report radiation-induced EGLS.^3^ Radiation therapy, or radiotherapy, is known for its effects on skin integrity and potentially inducing chronic inflammatory responses, which may have contributed to the local skin changes seen in our patient with EGLS.

Radiotherapy induces oxidative damage through generating reactive oxygen species, which directly causes DNA damage and toxic lipid peroxidation, diminishing cell survival.^4^ These effects overlap with the potential pathogenesis of EGLS, where oxidative stress is elevated within the dermis of LS lesions, suggesting that oxidative damage may contribute to the sclerosis and inflammation seen in EGLS.^5^

Interferon-gamma (IFN-γ), a cytokine involved in ionizing radiation therapy, plays an essential role in mediating desired antitumor effects.^6^ Increased IFN-γ staining in LS specimens was seen in 1 immunohistochemical study, suggesting that LS and chronic wounds may share similar cytokine responses.^7^ Further research should examine how IFN-γ contributes to the development of EGLS in postradiation patients. Radiotherapy-induced fibrosis results from chronic inflammation and oxidative damage, leading to reduced vascularity, excess collagen deposition, and scarring.

There are a couple of reports of EGLS in prior radiation fields. In 1 case, a 57-year-old Caucasian woman presented with a single bulla overlying an erythematous plaque on the right medial breast 5 months after radiation therapy for invasive ductal carcinoma. Histology showed bullous LS in the superficial dermis and morphea in the deep dermis, confirming radiation-induced morphea with a rare bullous variant. Initially treated with dicloxacillin for potential soft tissue infection, she returned 5 months later with bilateral breast involvement and worsening symptoms, including multiple hemorrhagic bullae and new, white, sclerotic regions. She was started on a 60 mg 20-day prednisone taper, then topical clobetasol 0.05% cream twice daily. After 2 months, she reported reduced discomfort and softening of the sclerotic areas, although new areas developed, requiring a second course of prednisone.^8^ This case of bullous LS required systemic corticosteroids for the later progression of the disease, while our case demonstrated a localized, nonprogressive EGLS lesion.

In a second case, a 77-year-old woman presented with left breast discomfort with ivory-white patches, overlying telangiectasia, and purpuric hemorrhages on physical exam. She had radiation therapy, breast-conserving surgery, and hormonal treatment for cancer of the left breast 2 years before the initial visit to dermatology. Biopsy confirmed LS, and betamethasone 0.05% cream led to some improvement. Pain and skin changes of the breast were still present after 6 months, and she elected to have a left mastectomy. The Koebner phenomenon is described to have triggered this patient’s LS at the site of radiation, which describes a potential mechanism for LS development in a radiation field.^9^

In another case, a 67-year-old woman presenting with a firm plaque in the perineal area was diagnosed 16 months prior with high-grade basaloid squamous cell carcinoma of the vagina, and treatment with radiation therapy and vaginal cuff brachytherapy was done. A biopsy confirmed the diagnosis of radiation-induced LS and was the first report of this in the vulvar region.^10^ While our case involved EGLS, this further supports the idea that radiation therapy may play a role in the pathogenesis of both genital and extragenital LS.

This case of EGLS in a postradiation field emphasizes the need for further research on the mechanisms linking EGLS with radiation therapy and the risk factors for its onset in postradiation patients.

## Conflicts of interest

None disclosed.
